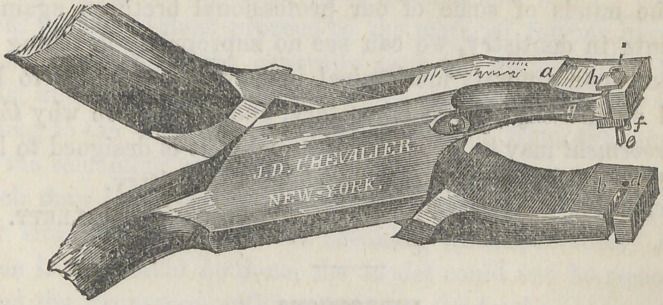# Plate Forceps

**Published:** 1856-12

**Authors:** Samuel Mallett


### PLATE FORCEPS.

This improvement consists in making an adjustable punch in such a manner that the pins in the mineral teeth will guide the punches so that they shall punch the holes in the plates to exactly correspond with the pins in the teeth.

The instrument is made in the ordinary form of pliers, as shown in the drawing.

The improvement includes the combination of two punches, one immovable, and the other movable in the slot ($h$) with a spring ($g$) and two cavities, one ($e$) in the plug or immovable punch, and the other ($f$) movable with the movable punch, the latter to be set by the inserting of the wires of the teeth into the cavities in order to make the distances between the holes in the plate correspond with the distance of the pins in the teeth as described.

To use this instrument we place one of the pins of the tooth into the hole (*f*) and force out the movable punch far enough to allow the other pin in the tooth to pass into the other hole (*e*) which will adjust the other two punches to exactly the distance of the pins, so that when the plate is punched, the holes in the plate will receive the pins with perfect accuracy in all cases.

The advantages of this instrument over the one in use is that it punches the holes for both pins at once, and that with great accuracy; if the pins in the teeth are straight, the punches being regulated in every case by the pins in the tooth.

2d. It may be used either for a double punch or as a single one, by removing the movable punch, which can be done almost instantly; we can change it from a double to a single one, and do away with the necessity of having any other in the laboratory.

This improvement is patented by Samuel Mallett and A. B. Smith, New Haven, Ct., and although there are prejudices in the minds of some of our professional brethren against patents in dentistry, we can see no impropriety in taking a patent upon a purely mechanical instrument, which is to be used exclusively in the laboratory, another reason why *this improvement* may be properly patented is, it is designed to be used for purposes other than mechanical dentistry.

SAMUEL MALLETT.

## Figures and Tables

**Figure f1:**